# Acoustoelectric brain imaging with different conductivities and acoustic distributions

**DOI:** 10.3389/fphys.2023.1241640

**Published:** 2023-10-31

**Authors:** Yijie Zhou, Xizi Song, Yibo Song, Jiande Guo, Gangnan Han, Xiuyun Liu, Feng He, Dong Ming

**Affiliations:** ^1^ Academy of Medical Engineering and Translational Medicine, Tianjin University, Tianjin, China; ^2^ College of Precision Instruments and Optoelectronics Engineering, Tianjin University, Tianjin, China

**Keywords:** acoustoelectric brain imaging, acoustoelectric imaging, acoustoelectric signal, conductivity, acoustic distribution

## Abstract

**Objective:** Acoustoelectric brain imaging (AEBI) is a promising imaging method for mapping brain biological current densities with high spatiotemporal resolution. Currently, it is still challenging to achieve human AEBI with an unclear acoustoelectric (AE) signal response of medium characteristics, particularly in conductivity and acoustic distribution. This study introduces different conductivities and acoustic distributions into the AEBI experiment, and clarifies the response interaction between medium characteristics and AEBI performance to address these key challenges.

**Approach:** AEBI with different conductivities is explored by the imaging experiment, potential measurement, and simulation on a pig’s fat, muscle, and brain tissue. AEBI with different acoustic distributions is evaluated on the imaging experiment and acoustic field measurement through a deep and surface transmitting model built on a human skullcap and pig brain tissue.

**Main results:** The results show that conductivity is not only inversely proportional to the AE signal amplitude but also leads to a higher AEBI spatial resolution as it increases. In addition, the current source and sulcus can be located simultaneously with a strong AE signal intensity. The transcranial focal zone enlargement, pressure attenuation in the deep-transmitting model, and ultrasound echo enhancement in the surface-transmitting model cause a reduced spatial resolution, FFT-SNR, and timing correlation of AEBI. Under the comprehensive effect of conductivity and acoustics, AEBI with skull finally shows reduced imaging performance for both models compared with no-skull AEBI. On the contrary, the AE signal amplitude decreases in the deep-transmitting model and increases in the surface-transmitting model.

**Significance:** This study reveals the response interaction between medium characteristics and AEBI performance, and makes an essential step toward developing AEBI as a practical neuroimaging technique.

## 1 Introduction

Brain activity is distributed over the 3D brain volume and evolves in time. To better understand the human brain working mechanism and study brain disease pathogenesis, it is crucial to non-invasively image brain dynamics with high spatiotemporal resolution. Due to the head volume conduction effect, electroencephalography (EEG) offers a high temporal resolution, but it has a limited spatial resolution to image brain activity (Berthon et al., 2019). With spatial focality and non-invasiveness, focused ultrasound (FUS) may be used to significantly enhance EEG spatial resolution (He, 2016). Acoustoelectric imaging (AEI) is an emerging technique that directly maps the biological current density distribution through the modulation of the tissue impedance induced by FUS (Olafsson et al., 2008). AEI has the potential to become a novel neuroimaging method, which is named acoustoelectric brain imaging (AEBI) (He, 2016; Zhang et al., 2022).

### 1.1 Acoustoelectric brain imaging

**FIGURE 1:**
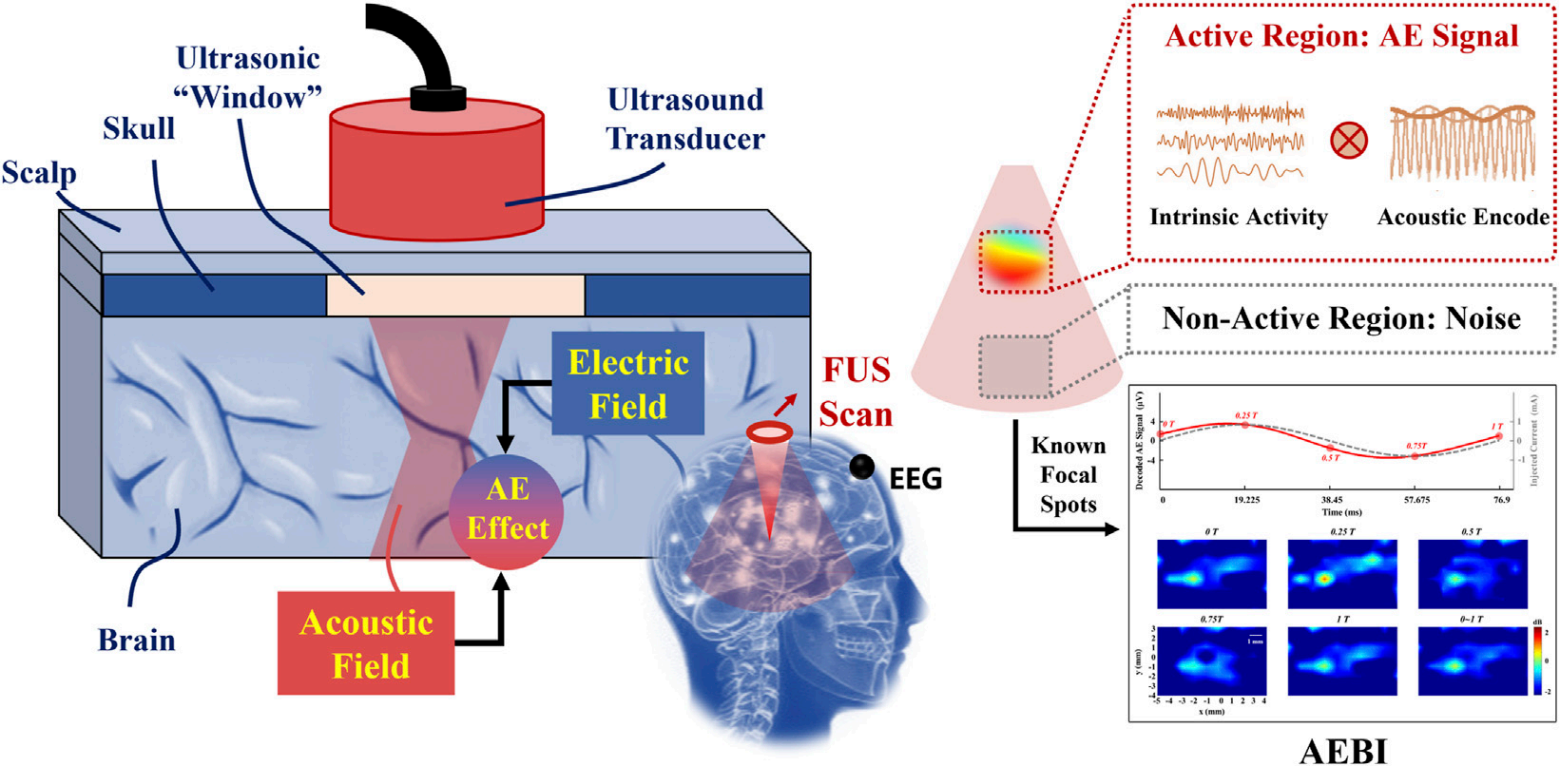
Schematic diagram of AEBI. The left panel zooms in on the focus inside. The right panel indicates AEBI process in the FUS scanning region.

Figure 1 shows the schematic diagram of AEBI. A transcranial FUS beam scans through the brain region of interest where the potential sources may be located, including the active and non-active regions. Based on the AE effect, the interaction between the acoustic and electric field will cause ultrasound encoded electric signals in the FUS-selected volume, which can be measured by EEG electrodes on the scalp. By scanning through the brain region of interest, the active region will generate a detectable high-frequency AE signal due to the coherent intrinsic activity encoded by FUS, whereas the non-active region will output noises due to incoherent activities (He, 2016). AE signals can be decoded to extract EEG in the highly focused brain volume that reflects the distribution of electrophysiological sources. Therefore, AEBI can be performed by AE signals at known focal spots. This approach promises to offer ms-level temporal and mm-level spatial resolution for human brain mapping from EEG.

Compared with conventional EEG, AEBI adopts an active imaging strategy to selectively focus on the target brain volume. AEBI gives EEG a specific ultrasonic spatial label to enhance spatial resolution without disturbing the original EEG. Localizing spatially by FUS, AEBI eliminates the need for solving the ill-posed EEG inverse problem which has been the major bottleneck in achieving high spatial resolution. In addition, AEBI still detects electrophysiological signals and does not involve multimodal information.

### 1.2 Related works

During ultrasound propagation in the medium, the local pressure changes cause periodic mechanical compression and expansion in the focused area. This small elastic deformation induces change in conductivity. Previous studies specifically focused on the study of AEI feasibility verification and method optimization. The first study (Olafsson et al., 2008) proposed AEI, a direct 3D imaging technique that potentially facilitates existing mapping procedures with superior spatial resolution. Wang et al. then demonstrated the AEI of a time-varying 3D current field based on simulation and phantom experiments (Wang et al., 2011; Wang and Witte, 2014). Recent studies performed AEI in rat, rabbit, and swine hearts to effectively localize the current density distribution (Qin et al., 2015; Berthon et al., 2019; Alvarez et al., 2020). In addition, some works have made a progress in improving AEI performance, such as sensitivity, spatial resolution, and signal-to-noise ratio (Qin et al., 2012; Wang et al., 2016). These studies showed that AEI is capable of high-resolution, volumetric current source density imaging with the potential for real-time electrical mapping at the millimeter and millisecond scales.

In recent years, efforts are being made to develop AEBI as a functional neuroimaging method. Some research workers concentrate on optimizing the AEBI hardware system. Berthon et al. proposed an ultrafast AEI system for the non-invasive ultrafast mapping of current densities (Berthon et al., 2017; Berthon et al., 2019). In the same year, Qin et al. designed a custom ultrasound array for 4-D (volume and time) AEBI with electronic beam-steering through the human skull (Qin et al., 2017). Then, Burton et al. (2018) developed a mobile AEBI platform to map the physiological activity in the rat hippocampus for easy transportation and adjustment. It is worth noting that some studies combine AEBI with specific brain disease diagnosis and treatment. For instance, AEBI was investigated as a new modality to non-invasively image and characterize the current produced from a directional deep brain stimulator lead (Preston et al., 2018; Preston et al., 2020). Another study employed a human head model with a real skull to demonstrate the feasibility of AEBI for the electrical mapping of deep dipole sources during the treatment of epilepsy with much better resolution and accuracy than that in conventional mapping methods (Barragan et al., 2020). Moreover, the source signal modulation mechanism of the pulse repetition frequency (PRF) of FUS by rat brain experiments was demonstrated, and the results showed that decoding EEG signals from the modulated AE signals is feasible (Zhou et al., 2019; Zhou et al., 2020a). Based on the PRF decoding method, it was proven that AEBI could be used to map living rat steady-state visual evoked potential activation, and the processing network of the AE signal in the living rat brain was revealed (Song et al., 2021; Song et al., 2022). The aforementioned studies are important steps toward optimizing AEBI imaging technology and translating it to clinical applications.

### 1.3 Contribution

AEBI is based on the AE effect, which is an interaction between electric and acoustic filed. It refers to the local conductivity change induced by acoustic pressure when the ultrasound beam is traversing the medium. Both conductivity and acoustic distribution of a medium are key factors that have effect on the AE signal, and then have great effect on AEBI through a spatially coding source signal by ultrasound. It is worth noting that skull and brain tissues have different conductivities and acoustic distributions. These medium differences may result in different AE signal responses, which needs to be fully considered for human AEBI.

In this work, we further explore the AEBI response to conductivity and acoustic distribution. The main content of this article is as follows:

• We conducted the AEI experiment, potential measurement, and simulation on the pig fat and muscle tissue to explore AEI performance and AE signal response with different conductivities. Then we further validated it in the AEBI experiment based on pig brain tissues with different sulcus distributions.
• We designed the deep and surface transmitting models for different acoustic distributions, using a human skull and pig brain tissue. Based on the AEBI experiment and acoustic field measurement, we explored and clarified AEBI performance and AE signal responses with different acoustic distributions.
• We combined the conductivity and acoustic distribution to comprehensively analyze the AE signal response interaction between medium characteristics and AEBI performances.

## 2 Theory

AE effect is the physical foundation of AEBI. Acoustic pressure change ∆*P* in response to wave propagating locally modifies the medium’s initial resistivity $\rho_{0}$ which causes resistivity change Δρ (Fox et al., 1946; Jossinet et al., 1998). It follows the equation

$$\Delta \rho =-\rho_{0}K\Delta P,$$ (1)

where *K* is a material-specific AE interaction constant which is in the order of 10^−9^ Pa^−1^ in 0.9% NaCl (Lavandier et al., 2000a). The local resistivity change produces the current modulation when electrical current passes through the medium.

A pair of electrodes is called a lead, and the sensitivity distribution of the lead is called a lead field. According to the reciprocity theorem, voltage $V_{i}$ measured by lead *i* is

$$V_{i}=∭\rho \left({\tilde{J}}_{i}^{L}\cdot J^{I}\right)dxdydz,$$ (2)

where $J^{I}=J^{I}\left(x,y,z\right)$ is the distributed current source and ${\tilde{J}}_{i}^{L}={\tilde{J}}_{i}^{L}\left(x,y,z\right)$ is the lead field of lead *i* (Malmivuo and Plonsey, 1995).

As ultrasound transmits a conducting medium, the resistivity distribution becomes

$$\rho =\rho_{0}-\rho_{0}K\Delta P.$$ (3)

Substituting (3) into (2) and replacing resistivity $\rho_{0}$ with conductivity $\sigma_{0}$ leads to

$$V_{i}={V_{i}}^{LF}+{V_{i}}^{AE},$$ (4)

$${V_{i}}^{LF}=∭\frac{1}{\sigma_{0}}\left({\tilde{J}}_{i}^{L}\cdot J^{I}\right)dxdydz,$$ (5)

$${V_{i}}^{AE}=∭\left(-\frac{1}{\sigma_{0}}K\Delta P\right)\left({\tilde{J}}_{i}^{L}\cdot J^{I}\right)dxdydz.$$ (6)

It is clear that $V_{i}$ is the summation of $V_{i}^{LF}$ (low-frequency EEG signal) and $V_{i}^{AE}$ (high-frequency AE signal). AE signal $V_{i}^{AE}$ is a band-pass filtered at high frequency (ultrasound center frequency or PRF) in the FUS-irradiated medium. Then, $V_{i}^{LF}$ and $V_{i}^{AE}$ can be split by frequency bands during or after acquisition (Olafsson et al., 2008). According to (6), the AE signal was directly proportional to the current density and also sensitive to the direction of the current flow (Lavandier et al., 2000b; Witte et al., 2007).

According to the aforementioned theory, ultrasound will periodically modify medium conductivity at that frequency (the center frequency or the PRF) when it scans near the dipole at a certain frequency. Then, the AE signal generated at the place with high current density is large, and it is relatively smaller at the place with low current density. Therefore, with the known focal spot and mm-level spatial resolution, AE signals can be obtained by scanning the region of interest and then used to map current densities.

## 3 Methods

This study was approved by the Ethics Committee of Tianjin University. An adult human cadaveric skull with its parietal cap removed and isolated fresh pig tissues were used in the experiment, which were provided by the Medical College of Tianjin University. The fresh pig tissues were stored under refrigeration at 4°C for 6 h after being euthanized from healthy long white pigs.

### 3.1 Experimental protocol for different conductivities

The AEI experiment, potential measurement, and simulation were conducted separately. To establish the dipole current fields with different conductivities and uniform distributions, a pig muscle and fat tissue were adopted in this experiment. The conductivity of the muscle and fat tissues were 10.78 mS/cm and 1.96 mS/cm measured by a conductivity meter, respectively.

#### 3.1.1 AE signal measurement for AEI

**FIGURE 2:**
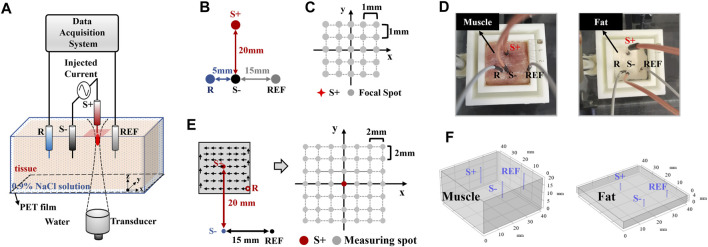
Experimental setup for different conductivities. **(A)** Overall AEI experimental diagram, **(B)** location of electrodes in the AEI experiment, **(C)** FUS scanning region of AEI, **(D)** the pig muscle and fat tissues used in this experiment, **(E)** electrode locations for conventional low-frequency measurement, and **(F)** the geometry of the current simulation.

Figure 2A shows the experimental setup for generating the dipole current field and detecting AE signals. To simulate a neural discharge, a 13-Hz sinusoidal current with the peak amplitude of 1 mA was injected through a pair of stimulating electrodes (marked “S+” and “S−”) into the muscle or fat tissue immersed in 0.9% NaCl solution. Figure 2B shows that the distance between S+ and S- was 20 mm. Recording electrode R was 5 mm to the left and reference electrode REF was 15 mm to the right of S-. To obtain the AE signal for mapping the current density, the ultrasound transducer was mechanically controlled to scan around current source S+ on the x–y plane, which is called C-scan in ultrasound imaging. At each focal spot, the AE signal was recorded simultaneously. Figure 2C shows that anode S+ of the dipole was defined as the origin of the rectangular coordinate system. With a 1-mm step in *x* and *y* directions, there were 5 × 5 focal spots in the scanning region. Figure 2D shows the muscle and fat tissue adopted in this experiment. To make medium conductivity the only variable under the same current source 1 mA, it is necessary to ensure that the measured voltage (the summation of low-frequency EEG signal and high-frequency AE signal) is comparable in the muscle and fat. According to Ohm’s law and conductivity ratio (muscle: fat ≈ 5:1), the resistance of fat should be reduced artificially to obtain the same voltage. Thus, the size (length × width × height) of the muscle and fat tissue was set to 40 mm × 40 mm × 20 mm and 40 mm × 40 mm × 4 mm, respectively, according to their conductivity ratio (muscle: fat ≈ 5:1). Then, a similar potential could be produced between recording electrode R and reference electrode REF for the muscle and fat tissues.

#### 3.1.2 Conventional low-frequency measurement

The dipole current field was independently measured using conventional methods to compare AEI and simulation results. Figure 2E shows that the potential was measured between a fixed-reference electrode REF and a mobile electrode R. Using the same tissue and current source to AEI, the potential distribution in the tissue was mapped with the mobile platinum electrode, which was mounted on a motorized 2-D translation stage, and scanned across a 6 × 6-step grid in the steps of 2 mm.

#### 3.1.3 Simulation of current distribution

To investigate the contribution of the current density, numerical simulation was implemented using COMSOL Multiphysics simulation software (AltaSim Technologies, Columbus, OH, United States). The finite-element method (FEM) was used to solve the stationary current problem. Figure 2F shows that the 3D geometry of 40 mm × 40 mm × 20 mm was built as the muscle tissue and the 3D geometry of 40 mm × 40 mm × 4 mm was built as the fat tissue, whose sizes are similar to the experimental condition. Electrical conductivity values were set as 10.78 mS/cm and 1.96 mS/cm for muscle and fat, respectively. A pair of cylinder-shape platinum electrodes was used for a 1-mA/m^3^ volume current injection. The relative locations of stimulating electrodes and the reference electrode on the *x–y* plane were also consistent with experimental settings. FEM and adaptive first-order triangular element mesh were used for the solver (Song et al., 2019). The simulation was implemented to further explore and explain the difference of current density distributions with different medium conductivities. As a stimulating current (a cylinder-shape electrode in a 3D electric field), its parameter should be set as the volume current in COMSOL. It means that 1 mA/m^3^ is the value of the simulated cylinder-shape volume current, instead of the current density of the cuboid-shape medium. Under the same value of stimulating current, it does not affect simulation results. Therefore, it can provide reference to the AEI and conventional measurement.

### 3.2 Experimental protocol for different acoustic distributions

To form the dipole current fields with different acoustic distributions, the deep-transmitting and surface-transmitting models were established on a human skullcap and a pig brain tissue for the AEBI experiment. For both models, a 13-Hz sinusoidal current with the peak amplitude of 0.5 mA was delivered between the stimulating electrode S+ and S-. During the current stimulation, the ultrasound beam was focused at the tip of the stimulating electrode S+ and was electronically scanned around S+ on the *x–y* plane to generate the AE signal for AEBI. Anode S+ of the dipole was defined as the origin of the rectangular coordinate system. With the 1-mm step in *x* and *y* directions, there were 5 × 5 focal spots in the scanning region. At each focal spot, the AE signal was recorded simultaneously from electrodes on the skullcap surface (extracranial, R1) and brain tissue (intracranial, R2).

It is worth noting that the ultrasound transmitting path was the main difference between the two models which was designed by changing the current source depth. For both models, the human skullcap was presoaked in 0.9% NaCl solution for >48 h to remove air bubbles and retain bulk skull conductivity closer to *in vivo* conditions. Whether or not there was a skullcap, the brain tissue was submerged in 0.9% saline during the experiment to retain closer to *in vivo* conditions.

#### 3.2.1 Deep-transmitting model

**FIGURE 3:**
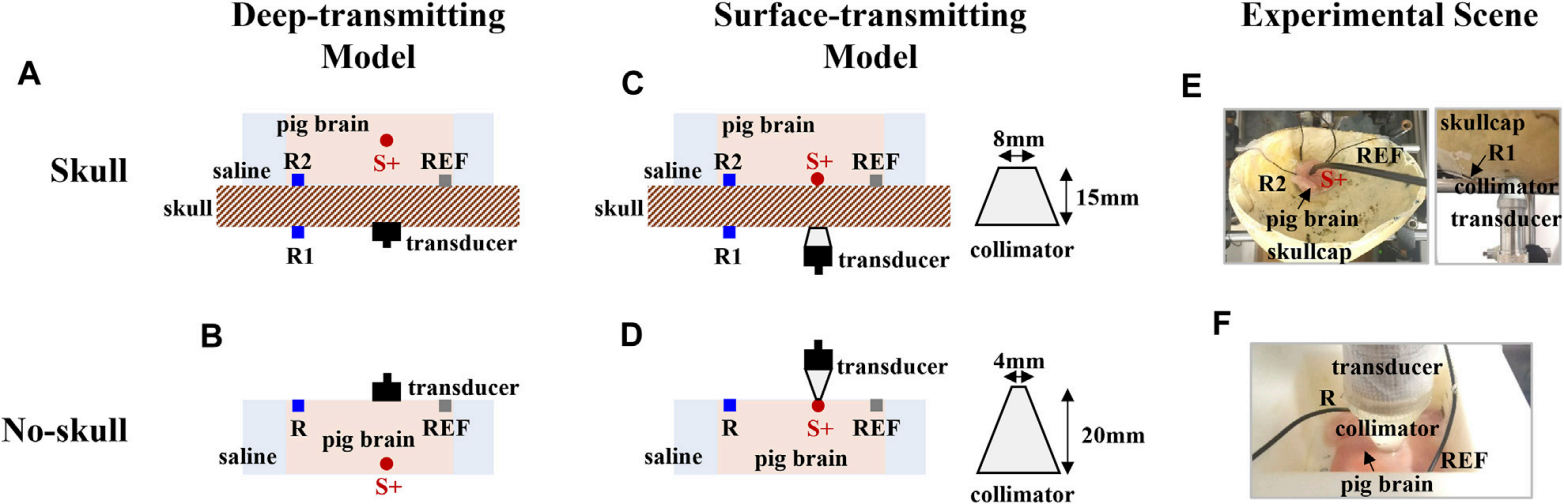
Experimental setup for different acoustic distributions. **(A)** and **(B)** Deep-transmitting model with and without the skull. **(C)** and **(D)** The surface-transmitting model with and without the skull. **(E)** and **(F)** The experimental scene of the surface-transmitting model with and without the skull.

Figures 3A, B show the deep-transmitting model with and without the skullcap, respectively. Figure 3A shows that the skullcap was placed under the brain tissue with stimulating electrode S+ embedded at a depth of 25 mm (focal length) above the skullcap’s outer surface for stimulation. For the no-skull condition shown in Figure 3B, the same brain tissue sample was placed in an imaging chamber filled with 0.9% NaCl solution. Stimulating electrode S+ was embedded in the brain tissue at a depth of 25 mm below the tissue surface. Recording electrode R and reference electrode REF were placed on the brain surface.

#### 3.2.2 Surface-transmitting model

Figures 3C, D show the surface-transmitting model with and without the skullcap, respectively. Figure 3C shows that the skullcap was placed under the brain tissue with platinum needle electrode S+ embedded at the interface between the tissue and skullcap for stimulation. According to the 5-mm thickness of the skullcap, a 15-mm-long collimator was adopted so that the distance between S+ and transducer was exactly the focal length 20 mm. For the no-skull condition shown in Figure 3D, the same brain tissue sample was placed in an imaging chamber filled with 0.9% NaCl solution. Stimulating electrode S+ and both recording electrode R and reference electrode REF were both placed on the brain surface. Another 20-mm-long collimator was adopted to achieve the same ultrasound transmitting distance. We consider the surface-transmitting model as an example, and the experimental scene is shown in Figures 3E, F.

### 3.3 Description of instrumentation

Excited by the ultrasonic pulser/receiver (5077PR, Olympus, JP), a single-element focused transducer (Olympus, A303S, 1.0 MHz, 20 mm focal length) was coupled to the skullcap with acoustic gel. The transducer was mounted onto a three-axis stage with the stepper motor of the 3D motion control (WNMC400-300B, Winner Optical Instruments Group Company Ltd, CHN) to implement an ultrasonic scan. The stimulating current waveform was generated by the programmable current output function in a source measurement unit (PXIe-4162, National Instruments, United States). Platinum needle electrodes (0.5 mm in diameter) were used for current stimulation and signal recording. Ag/AgCl cylindrical electrodes (1.0 mm in diameter) were adopted to record the AE signal in the AEBI experiment. The AE signal was acquired, amplified, and filtered by the SynAmps2 system (Neuroscan, United States) with a sampling rate of 20 kHz, and the band-pass filtering range was 0–3500 Hz. It has been proven that the high-frequency AE signal is band-pass filtered by the ultrasound center frequency or pulse repetition frequency (PRF) in the FUS-irradiated medium (Olafsson et al., 2008; Zhou et al., 2020b). In this study, PRF (1 kHz) was used to extract the AE signal from the raw signal, and the voltage signal was acquired with a 20-kHz sampling rate which has been validated in the previous work (Zhou et al., 2019; Zhou et al., 2020a; Zhou et al., 2020b; Song et al., 2021; Song et al., 2022; Zhang et al., 2022). In the AEBI experiment with different acoustic distributions, the pulse echo signal was collected by an NI PXI-5105 acquisition card (National Instruments, United States) at a 5-MHz sampling rate for further acoustic analysis.

**FIGURE 4:**
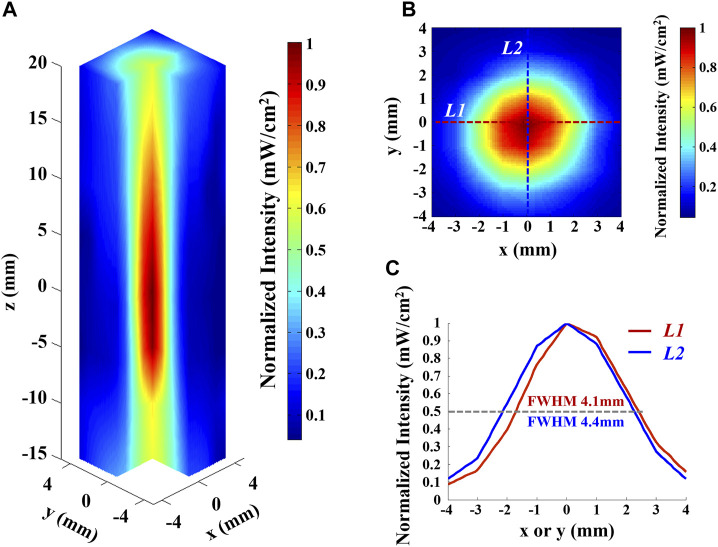
Acoustic intensity field of the ultrasound transducer used in this study. **(A)** Three-dimensional hydrophone scan of the ultrasound field of the transducer in water, normalized to maximum intensity. **(B)** Intensity profile in the plane orthogonal toward the axis and at the axial distance of maximum intensity (*z* = 0 mm), normalized to maximum. **(C)** Normalized intensity profile along *L1* and *L2* marked in **(B)**. Gray dashed line indicates the 50% intensity mark.

Before the experiment, the acoustic intensity field of the ultrasound transducer was measured by a hydrophone in the ultrasonic focal domain and surrounding areas within the water body, as shown in Figure 4, where Figure 4A shows the 3D distribution of the ultrasonic focal domain, Figure 4B shows the *XY* cross-section of the acoustic field at the axial distance of maximum intensity (*z* = 0 mm), and Figure 4C shows the acoustic intensity profile along *L1* and *L2* marked in the *XY* cross section. The ultrasound focus distributes with a half-maximal intensity diameter of 4.1 mm in the *x* direction and 4.4 mm in the *y* direction. The adopted ultrasound focus is of mm-level spatial resolution. Determined by the size of the focal spot, the spatial resolution of AEBI can reach to millimeter.

### 3.4 Imaging protocol and signal processing

#### 3.4.1 Imaging protocol

In this study, PRF was used to extract the AE signal from the raw signal for AEI which has been validated in the previous work (Zhou et al., 2019; Zhou et al., 2020a; Zhou et al., 2020b; Song et al., 2021; Song et al., 2022; Zhang et al., 2022). The acquired raw data were three-dimensional: C-scan sample points × *x*-location × *y*-location. For each sample point, the raw signal was downsampled at 5000 Hz and was band-pass filtered between PRF±50 Hz with a Butterworth filter. As a result, the AE signal was obtained. Then, the AE signal was converted to a complex form by the Hilbert transform. By calculating the absolute value of the complex form AE signal, its envelope (decoded AE signal) was obtained, which determined the magnitude of the local current densities. The 60-s averaged value of envelope was calculated as the AE signal amplitude of each sample point. According to the known coordinate of each sample point, the corresponding matrix of the AE signal amplitude could be formed. Finally, the corresponding matrix was interpolated at a 0.01 interval to reconstruct the AE image on a hot color map, indicating the intensity of local current densities.

#### 3.4.2 AE signal response analysis

For the AE signal response with different conductivities, the AE signal amplitudes of all sample points were compared using an independent-sample *t*-test. For different acoustic distributions, the AE signal amplitude was analyzed by a violin diagram to compare AE response intensities. To further analyze the AE signal response in the time domain, Pearson’s correlation coefficient was calculated to indicate the linear correlation degree of the decoded AE signal and source signal. The frequency–amplitude spectrogram of the decoded AE signal was calculated *via* fast Fourier transform (FFT). Based on the frequency–amplitude spectrum, FFT-SNR was defined as the ratio of FFT amplitude to the mean value of the 10 neighboring frequencies (i.e., five frequencies on each side), which was described by the frequency-SNR spectrum (Chen et al., 2015). According to the FFT-SNR value at source frequency 13 Hz of each sample point, the corresponding 13 Hz-FFT-SNR spatial distribution was further mapped, which reflected the AE signal response in frequency–spatial dimensions.

## 4 Results

### 4.1 AEI with different conductivities

**FIGURE 5:**
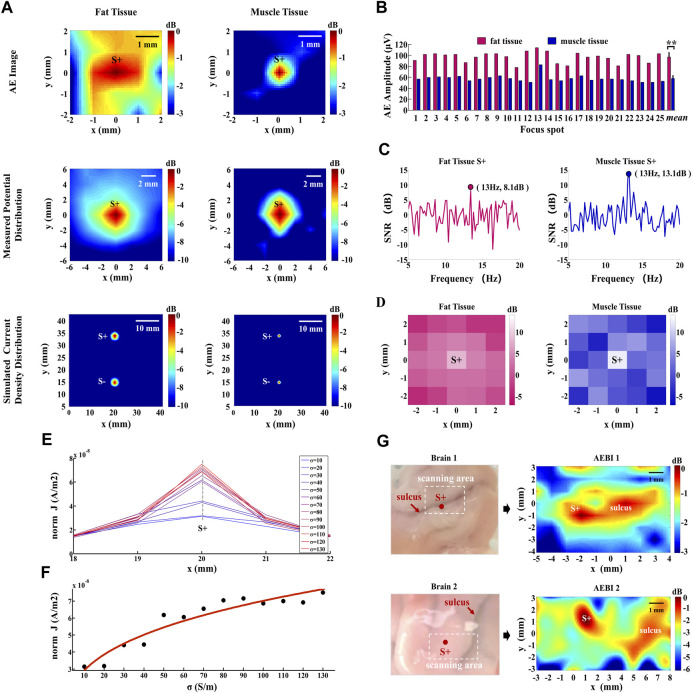
AEI and AE signal response with different conductivities. **(A)** AE images, measured potential distribution, and simulated current density distributions of fat and muscle tissues. Hot colors represent the magnitude of AE intensities, potentials, and current densities with a 3- or 10-dB dynamic range. **(B)** AE signal amplitudes at all focal spots of fat and muscle tissues. Error bar indicates the standard deviation, *represents the significant difference, *p < 0.05, **p < 0.01. **(C)** FFT-SNR of the decoded AE signal at the current source S+ (*x* = 0, *y* = 0) for fat and muscle tissues. **(D)** 13 Hz-FFT-SNR mapping of the decoded AE signal at all focal spots. Hot colors represent the value of 13 Hz-FFT-SNR from −3 dB to 14 dB. **(E)** Simulated current densities along the *x* direction for different conductivity values. **(F)** Curve fitting of current densities and conductivities along the gray dashed line marked in **(E)**. **(G)** AEBI tests of pig brain tissues with different sulcus distributions.

The 2D images formed from AE signals, the conventional low-frequency measurement, and the simulated current density are shown in Figure 5A. As displayed in the AE images of fat and muscle tissues, the focal spot (*x* = 0, *y* = 0) can be located as the current source S+ with a stronger AE signal intensity. With higher conductivity, the muscle tissue reaches a relatively higher AEI spatial resolution than the fat tissue. This spatial resolution difference between fat and muscle can also be observed in measured potential and simulated current density distributions. These results demonstrate that AEI can effectively map the current field with different conductivities. Moreover, AEI shows a higher spatial resolution with higher medium conductivity due to the original distribution of the current density.

### 4.2 AE signal response of different conductivities

#### 4.2.1 Amplitude analysis

To explore the AE response intensity with different conductivities, the AE signal amplitude is analyzed for all focal spots in fat and muscle tissue. Figure 5B shows that the AE signal amplitude of fat is stronger than that of muscles at each focal spot. Their averaged amplitudes are, respectively, 97.6 μV and 58.3 μV. According to the result of the independent-sample *t*-test, there is significant difference between the AE signal amplitude of fat and muscle tissues: *t*(24) = 23.09, *p* < 0.001. This indicates that the AE signal amplitude decreases with higher medium conductivity.

#### 4.2.2 Frequency-spatial analysis

To confirm the frequency feature of the decoded AE signal at S+ (*x* = 0, *y* = 0), the FFT-SNR and its spatial mapping are further analyzed for fat and muscle tissues. FFT-SNR (in decibels) of decoded AE signals from 5 Hz to 20 Hz is shown in Figure 5C. It can be observed that higher SNRs of decoded AE signals appear at the current source frequency of 13 Hz for both fat (8.1 dB) and muscle (13.1 dB) tissues. Furthermore, the 13-Hz-FFT-SNR spatial distribution was mapped by the SNR value at 13 Hz of each focal spot. Figure 5D shows that the maximum 13-Hz-FFT-SNR appears at current source S+ for both fat and muscle tissues. With a higher 13-Hz-FFT-SNR in muscle tissues, the value also decreases faster around current source S+ so that its spatial distribution shows a higher spatial resolution, which is consistent with an AE image. These results indicate that the SNR of the decoded AE signal at the current source frequency increases with higher medium conductivity and its spatial distribution is consistent with the AE image.

### 4.3 Analysis of conductivities

According to the results of AEI and AE signal responses with different conductivities shown in Figures 5A–D, the differences mainly exist in the AEI images and AE signal amplitudes. The difference of AEI spatial resolution is mainly caused by distributed current source $J^{I}$ . Thus, (6) can be written as

$${V_{i}}^{AE}\left(\sigma_{0}\right)=∭\left(-\frac{1}{\sigma_{0}}K\Delta P\right)\left({\tilde{J}}_{i}^{L}\cdot J^{I}\left(\sigma_{0}\right)\right)dxdydz.$$ (7)

Equations 6, 7 emphasize the role of conductivity by writing ${V_{i}}^{AE}\left(\sigma_{0}\right)$ and $J^{I}\left(\sigma_{0}\right)$ to describe the mathematical relation between conductivity and AE signals, respectively. As described in (7), conductivity has effect on AEI and AE signal amplitudes by $J^{I}\left(\sigma_{0}\right)$ and $\frac{1}{\sigma_{0}}$ , respectively. As a constant coefficient of $\frac{1}{\sigma_{0}}$ on the right side of (7), the AE signal amplitude is inversely proportional to conductivity. This relation effectively explains that the AE signal amplitude decreases with an increasing medium conductivity. However, based on conductivity values of fat and muscle tissues, the relation between conductivity $\sigma_{0}$ and current density $J^{I}\left(\sigma_{0}\right)$ is still not clear.

Therefore, current density $J^{I}\left(\sigma \right)$ with more conductivity values (= 10, 20, 30, 40, 50, 60, 70, 80, 90, 100, 110, 120, and 130 S/m) is further simulated on a 2D model. Centered on current source S+, the normalized value of current density $J^{I}\left(\sigma \right)$ along the *x* direction is shown in Figure 5E. It can be observed that the current density decreases gradually from current source S+ to both sides. In addition, as conductivity increases, the current density at S+ (*x* = 20 mm) also increases non-linearly. With higher conductivity, the current density decreases faster within the same spatial range, which results in higher AEI spatial resolution. Along the gray dashed line marked in Figure 5E, the current density value of each conductivity is further used for curve fitting. Figure 5F shows that the fitting curve (red solid curve) calculated from discrete data (black dots) can be described as

$$\left|J_{source}^{I}\left(\sigma \right)\right|=1.809\times 10^{-8}\times \sigma^{0.3196}-8.623\times 10^{-9}.$$ (8)

The unit is S/m for conductivity and A/m^2^ for current density. The fitting goodness is evaluated by the sum of the squared errors 2.529 × 10^−16^, R-square 0.9117, and root mean square error 5.029 × 10^−9^, presenting an effective fitting ability.

In this section, the extensive AEBI experiments were conducted to further confirm the effect of conductivities on AEI. In consideration of the conductivity difference in the brain (gray matter: 0.2917 S/m and white matter: 0.1585 S/m) and cerebrospinal fluid (2.002 S/m) which exists in the sulcus, two pig brain tissues with different sulcus distributions were used for AEBI tests. The brain tissues (brain 1 and brain 2) and the corresponding AEBI results (AEBI 1 and AEBI 2) are shown in Figure 5G, marked with the scanning region and current source S+ in the left panel. For brain 1, the sulcus is distributed laterally and current source S+ is located in the area of the sulcus. As displayed in the AEBI 1 image, current source S+ and the sulcus around S+ can be located simultaneously with strong AE signal intensity. Different from brain 1, the sulcus of brain 2 is distributed vertically and current source S+ is distant from the sulcus. As shown in the AEBI 2 image, current source S+ and the distant sulcus also can be located simultaneously. These results indicate that the sulcus with higher conductivity can be imaged by a stronger AE signal response which further validates the effect of conductivity on AEBI.

### 4.4 AEBI with different acoustic distributions

**FIGURE 6:**
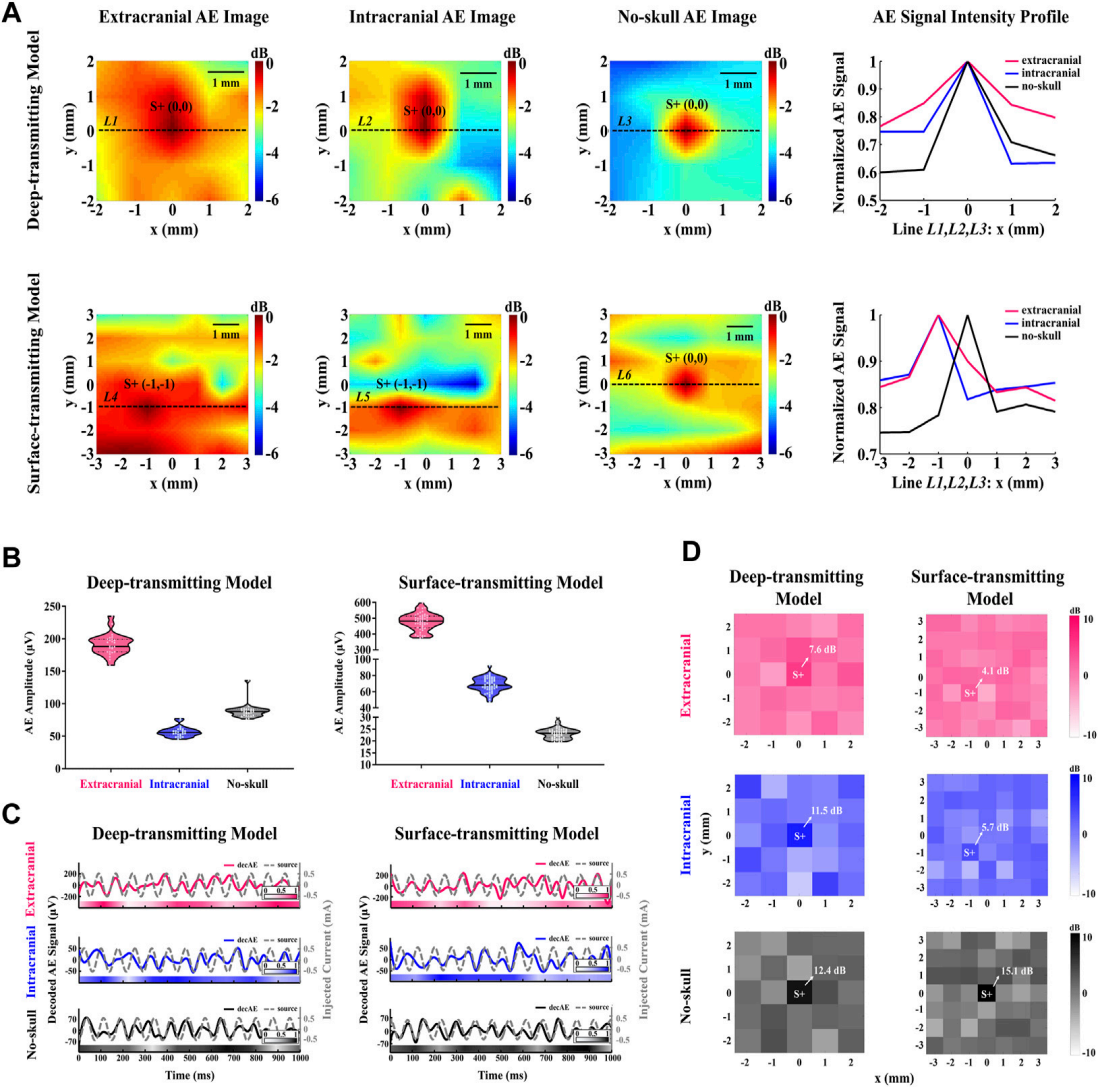
AEBI and AE signal response with different acoustic distributions. **(A)** Extracranial, intracranial, and no-skull AE images of deep and surface transmitting models. Hot colors represent the magnitude of AE intensity with a 6-dB dynamic range. AE intensity profile along the black dashed line L1 to L6 marked in AE images, normalized to maximum intensity. **(B)** Extracranial, intracranial, and no-skull AE signal amplitudes of deep and surface transmitting models. White dots represent AE signal amplitudes at each focal spot and horizontal solid lines represent the median. **(C)** Extracranial, intracranial, and no-skull waveforms of the decoded AE signal (red, blue, and black solid curve) and the source signal (gray dashed curve) for deep and surface transmitting models. The left and right scales are for the decoded AE signal and source signal, respectively. The correlation between decoded AE signal and source signal is indicated by the color bar along the time axis whose value refers to the color bar in the legend. **(D)** Extracranial, intracranial, and no-skull 13 Hz-FFT-SNR mapping of the decoded AE signal for deep and surface transmitting models. Hot colors represent the value of 13 Hz-FFT-SNR from −10dB to 10 dB.

Figure 6A shows that current source S+ with strong AE signal intensity can be located in the extracranial, intracranial, and no-skull AE images. For the deep-transmitting model, current source S+ is located (*x* = 0, *y* = 0) in each AE image, which is consistent with the stimulating electrode position. For the surface-transmitting model, current source S+ is located at (*x* = −1, *y* = −1), (*x* = −1, *y* = −1), and (*x* = 0, *y* = 0) in the extracranial, intracranial, and no-skull AE images, respectively. The located deviation of current source S+ can be observed in extracranial and intracranial AE images likely due to the transcranial ultrasound field change. In addition, the AEBI spatial resolution is evaluated by the AE signal intensity profile at current source S+ along the *x* direction. As shown in the right panel of Figure 6A, each curve exhibits that AE signal intensity decrease from current source S+ to both sides. There is a decreasing slope in order of the no-skull, intracranial, and extracranial AE images. This indicates that there is a loss in AEBI spatial resolution (1–4 mm) likely due to the ultrasound field change and electrical impedance mismatch between the tissue and skullcap.

### 4.5 AE signal response of different acoustic distributions

#### 4.5.1 Amplitude analysis

The AE signal amplitude is analyzed by violin diagrams to compare the extracranial, intracranial, and no-skull AE response intensities in the deep and surface transmitting models. Figure 6B shows AE signal amplitudes at all focal spots described by white dots in red, blue, and black violin corresponding to the extracranial, intracranial, and no-skull AEBI. For the deep-transmitting model, extracranial AEBI shows high AE response intensity with the averaged value of 191.08 μV. The intracranial AEBI (averaged 56.66 μV) appears to have lower amplitude than that of no-skull (averaged 88.64 μV). Compared with the intracranial and no-skull AEBI, extracranial AEBI shows a much stronger intensity and its AE signal amplitudes are relatively more discrete. For the surface-transmitting model, the extracranial AEBI also shows high AE response intensity with averaged 477.00 μV, which is consistent with that of a deep-transmitting model. Different from the deep-transmitting model, the intracranial AEBI (averaged 68.67 μV) appears to have higher amplitude than that of no-skull (averaged 23.28 μV). In addition, extracranial and intracranial AEBI shows relatively more discrete AE signal amplitudes than no-skull. These results indicate that AE signal amplitudes of the extracranial, intracranial, and no-skull AEBI change differently in the deep and surface transmitting models.

#### 4.5.2 Timing analysis

To further demonstrate the AE signal response in the time domain, the waveform of the decoded AE and source signals is exhibited in Figure 6C. A total of 13 cycles (1 s) of the periodic signal are displayed for the extracranial, intracranial, and no-skull AEBI of deep and surface transmitting models. For each condition, a clear periodicity is observable for each decoded AE signal, matching the frequency of the simultaneously measured source signal. In addition, the decoded AE and source signals are positively correlated in both amplitudes and phase angles. The correlation between decoded AE and source signals is indicated by the color bar along the time axis. For the deep-transmitting model, the correlation analysis yields a significant positive relationship between decoded AE and source signals (extracranial: *r* = 0.36, *p* < 0.001; intracranial: *r* = 0.39, *p* < 0.001; and no-skull: *r* = 0.52, *p* < 0.001). For the surface-transmitting model, the correlation analysis also yields a significant positive relationship between source and decoded AE signals (extracranial: *r* = 0.28, *p* < 0.001; intracranial: *r* = 0.37, *p* < 0.001; and no-skull: *r* = 0.61, *p* < 0.001). These results show that the timing response of the decoded AE signal decreases gradually when recording from the no-skull to extracranial AEBI.

#### 4.5.3 Frequency-spatial analysis

Considering the decoded AE signal at the current source shows a good timing performance, the 13-Hz-FFT-SNR spatial distribution is further analyzed to confirm its frequency response in space. Figure 6D shows that the maximum 13-Hz-FFT-SNR appear at current source S+ for both deep (extracranial: 7.6 dB; intracranial: 11.5 dB; and no-skull: 12.4 dB) and surface (extracranial: 4.1 dB; intracranial: 5.7 dB; and no-skull: 15.1 dB) transmitting models. The value of 13-Hz-FFT-SNR decreases gradually from no-skull to extracranial AEBI. As a result, the 13-Hz-FFT-SNR mapping shows a decreasing spatial resolution in order of the no-skull, intracranial, and extracranial, which is consistent with AEBI images.

### 4.6 Analysis of acoustics

#### 4.6.1 Deep-transmitting model

**FIGURE 7:**
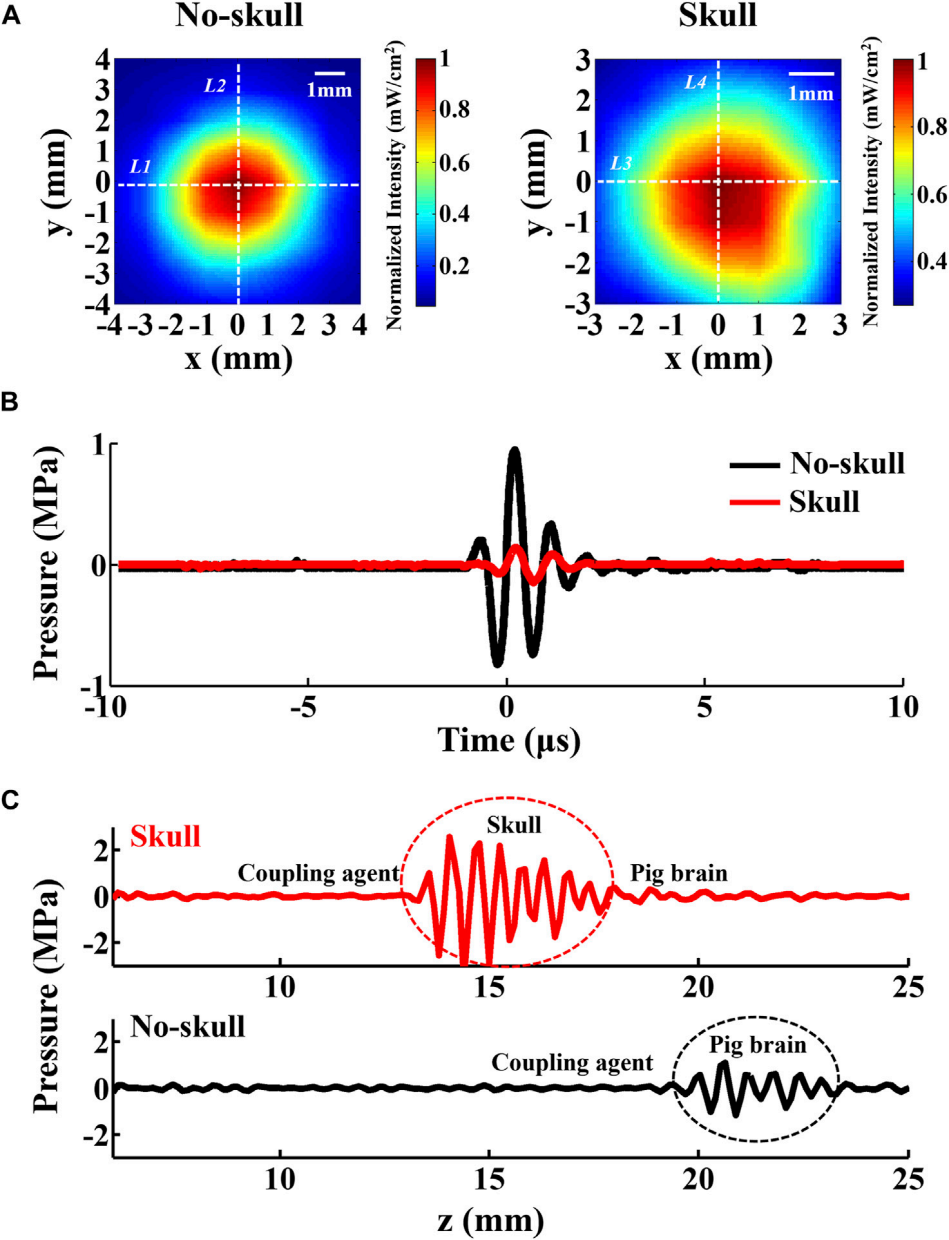
Effect of the skull on the focal zone and ultrasound pressure. **(A)** Ultrasound field distribution of the transducer in 0.9% NaCl solution with and without the skull, normalized to maximum intensity. **(B)** Comparison between the recorded pressure signal at the focus shown in **(A)**. **(C)** Comparison between the pulse echo signal with and without the skull in the surface-transmitting model. The transducer face is at *z* = 0 mm.

Ultrasonic transmission in the skull is the only variable between the intracranial and no-skull AEBI of the deep-transmitting model, which might cause different AEBI performance. The skull has a stronger ultrasound absorbing ability than the brain, with the absorption coefficient of 13 dB/cm (skull) and 0.9 dB/cm (brain). Therefore, ultrasound pressure attenuation caused by skull absorption and corresponding acoustic field change needs further exploration for the AEBI of the deep-transmitting model. The ultrasound field distribution of the used focus ultrasound with and without skull is displayed in Figure 7A, which is measured by a hydrophone in 0.9% NaCl solution. Defined with the full width at half maxima (FWHM), the dimensions of skull and no-skull are (*x* = 4.1 mm and *y* = 4.4 mm) and (*x* = 5.3 mm and *y* > 5.4 mm), which is calculated by white dashed lines (skull: *L1*, *L2* and no-skull: *L3*, *L4*) marked in Figure 7A. The focal zone with the skull is 1.67 times the size of no-skull, which appears to have a weakened focus performance. At the same time, there is an effect of a thick skull on ultrasound pressure. Figure 7B shows that the attenuation of the pressure wave through a 5-mm segment of the skull was 80% at focus. These results indicate that the focal zone enlargement and pressure attenuation with the skull might produce effects on AEBI which are further discussed with conductivity in the next section.

#### 4.6.2 Surface-transmitting model

In the surface-transmitting model, current source S+ was embedded at the interface between the brain tissue and skullcap (with skull) or on the brain surface (no skull). To confirm the difference of ultrasound echo, the pulse echo signal at current source S+ was acquired by the transducer during the AEBI experiment. Figure 7C shows that the red and black waves represent pulse echo with and without the skull, respectively. Evidently, there is a strong echo (marked by red circle) appearing at the interface between skullcap and brain tissues, which is approximately 2.3 times stronger than that of no-skull (marked by a black circle). Due to different acoustic impedances of the skull (6.184 × 10^6^ Pa s/m) and the brain (1.51 × 10^6^ Pa s/m), reflection and scattering occur when ultrasound waves arrive at the interface of the skull and the brain, causing an enhanced echo. This result indicates that the ultrasound echo enhancement with the skull might produce effect on AEI which is further discussed with conductivity in the next section.

## 5 Discussion

### 5.1 AEBI with different conductivity distributions

According to the AE effect and AE signal described in theory, conductivity has great effect on the AE signal and AEI. To confirm AEI performance and AE signal response with different conductivities, we performed the AEI experiment, potential measurement, and simulation on pig fat and muscle tissues. The results indicate that conductivity directly affects the AE signal amplitude and they are in inverse proportion. In addition, conductivity also affects AEI by changing the current density at the current source, which leads to a higher AEI spatial resolution with increasing conductivity.

To further confirm the aforementioned conclusion in AEBI, two pig brain tissues with different conductivity distributions were used for AEBI tests. The results validate that current source S+ and the sulcus can be located simultaneously and the sulcus shows a stronger AE signal response than other non-source S+ areas. In the sulcus with high conductivities, the current density is stronger than that of non-source S+ areas. Due to this difference of current density distribution, a stronger AE signal response appears in the sulcus which is distant from current source S+. In addition, current source S+ and the sulcus area can be distinguished by AE signal response features such as intensity, spatial resolution, and timing correlation. AEBI not only can be used for functional brain imaging but also shows potential in structural imaging.

### 5.2 AEBI combined with conductivity and acoustics

In this study, experimental models with different acoustic distributions were established on the human skullcap and pig brain tissues. It was natural to introduce the conductivity differences of mediums into AEBI experiments. Both the conductivity and acoustic distributions are basic physical properties of mediums and play an important role in AEBI. Therefore, it is critical to combine conductivities and acoustics to discuss the effect of the medium on AEBI.

For the deep-transmitting model (intracranial AEBI vs*.* no-skull AEBI), AEBI performance is probably affected by the focal zone enlargement and pressure attenuation caused by skull absorption. According to AE signal formula described in (6), the integral has the opposite effect on the AE signal amplitude and spatial resolution. Particularly, the focal zone enlargement leads to an increased integral domain. As a result, the AE signal amplitude increases with the loss of spatial resolution and other AEBI performances, such as 13 Hz-FFT-SNR and timing correlation. Different from focal zone enlargement, the pressure attenuation makes the AE signal amplitude decrease. Except for the acoustic effect, it is essential to take conductivity into account for AEBI analysis due to its difference in the skull (0.04282 S/m) and the brain (gray matter: 0.2917 S/m and white matter: 0.1585 S/m). Because the conductivity of the skull is approximately 15–27% of the brain tissue, the whole conductivity of the skull–brain used in intracranial AEBI is much lower than that of the brain tissue used in no-skull AEBI. Compared with no-skull AEBI, this also makes the AE signal amplitude increase and the imaging performance decrease for the intracranial AEBI. Under the comprehensive effects of conductivities and acoustics, the intracranial AEBI finally shows decreased AE signal amplitude and reduced AEBI performance by contrast with no-skull AEBI.

For the surface-transmitting model (intracranial AEBI vs*.* no-skull AEBI), the ultrasound echo enhancement caused by both the reflection and decreasing conductivities of the skull–brain (intracranial) contributes to the increased AE signal amplitudes. In addition, the intracranial AEBI performance (spatial resolution, FFT-SNR, and timing correlation) is also affected by the focal zone enlargement and decreased conductivities, which is similar to the deep-transmitting model. Compared with the deep-transmitting model, there is a 13-Hz-FFT-SNR loss at current source S+ in the extracranial and intracranial AEBI of the surface-transmitting model. It is probably related to the ultrasound scattering at the interface between the skullcap and brain, leading the ultrasound energy to a new transmitting path.

To map current densities around the current source, the AE signals from the *x–y* plane of the current source were generated by C-scan of ultrasound, which is primary for the analysis of AEBI performance and AE signal response. For future research, the AE signal in the depth direction should be further explored, which is also indispensable for the comprehensive understanding of AEBI.

## 6 Conclusion

In this study, AEBI with different conductivities and acoustic distributions were realized and the AE signal response were analyzed effectively from multiple dimensions. Based on these results, the response interaction between medium characteristics and AEBI performance was revealed. Furthermore, the simultaneous effect of medium conductivity and acoustics was comprehensively discussed, which is of great significance to the practical application of AEBI. This study makes an essential step toward developing AEBI as a practical neuroimaging technique and provides theory guidance for further AEBI research.

## Data Availability

The original contributions presented in the study are included in the article; further inquiries can be directed to the corresponding authors.
